# Regulation of Immune Function by the Lymphatic System in Lymphedema

**DOI:** 10.3389/fimmu.2019.00470

**Published:** 2019-03-18

**Authors:** Raghu P. Kataru, Jung Eun Baik, Hyeung Ju Park, Itay Wiser, Sonia Rehal, Jin Yeon Shin, Babak J. Mehrara

**Affiliations:** Division of Plastic and Reconstructive Surgery, Department of Surgery, Memorial Sloan Kettering Cancer Center, New York, NY, United States

**Keywords:** lymphatic vessels, immune function, Th2 type T cells, inflammation, fibrosis

## Abstract

The lymphatic vasculature has traditionally been thought to play a passive role in the regulation of immune responses by transporting antigen presenting cells and soluble antigens to regional lymph nodes. However, more recent studies have shown that lymphatic endothelial cells regulate immune responses more directly by modulating entry of immune cells into lymphatic capillaries, presenting antigens on major histocompatibility complex proteins, and modulating antigen presenting cells. Secondary lymphedema is a disease that develops when the lymphatic system is injured during surgical treatment of cancers or is damaged by infections. We have used mouse models of lymphedema in order to understand the effects of chronic lymphatic injury on immune responses and have shown that lymphedema results in a mixed T helper cell and T regulatory cell (Treg) inflammatory response. Prolonged T helper 2 biased immune responses in lymphedema regulate the pathology of this disease by promoting tissue fibrosis, inhibiting formation of collateral lymphatics, decreasing lymphatic vessel pumping capacity, and increasing lymphatic leakiness. Treg infiltration following lymphatic injury results from proliferation of natural Tregs and suppresses innate and adaptive immune responses. These studies have broad clinical relevance since understanding how lymphatic injury in lymphedema can modulate immune responses may provide a template with which we can study more subtle forms of lymphatic injury that may occur in physiologic conditions such as aging, obesity, metabolic tumors, and in the tumor microenvironment.

## The Lymphatic System Directly and Indirectly Regulates Immune Responses

The lymphatic system is comprised of a series of blind ended, single cell thick initial lymphatic vessels that drain progressively into successively larger vessels and eventually return interstitial fluid back to the systemic circulation. Lymphatic flow is regulated by coordinated pumping of smooth muscle cells that partially envelop collecting lymphatics and compressive forces from surrounding skeletal muscles. One-way valves in collecting lymphatics ensure forward flow of interstitial fluid and prevent reflux when a segment of the collecting vessel located between two valves contracts (1).

In addition to draining interstitial fluid, the lymphatics system is responsible for lipid and fatty acid absorption and is an important regulator of cholesterol metabolism (2). The lymphatic system also regulates immune responses by transporting bacteria, foreign antigens, particulate matter, exosomes, and immune cells to regional lymph nodes and lymphoid structures (3). Regulation of immune responses occurs at multiple levels and is both active and passive in nature. Active mechanisms of immune response regulation by the lymphatics includes regulation of immune cell entry and migration through the lymphatic system by LEC cytokine, chemokine, and adhesion molecule expression. In addition, LECs modulate immune responses and regulate autoimmunity by transferring self-antigen to DCs (4), or by directly inducing T cells tolerance using their PD-L1 molecule or MHC II-self antigen peptide complex that acquired from DCs (5–7). The lymphatic system can also control immune responses indirectly by modulating the rate at which antigens and cells are delivered to regional lymph nodes by regulating lymphatic vessel tone and pumping (8–11).

Given the important role of the lymphatic system in a wide range of physiologic processes, it is not surprising therefore that abnormalities in lymphatic function have been implicated in inflammatory disorders (12, 13), immune tolerance (14), metabolic abnormalities such as obesity and metabolic syndrome (2), cardiovascular disease including hypertension and atherosclerosis (15), cancer growth and metastasis (16–18), infectious diseases (19, 20), and septic shock (21). Genetic, iatrogenic, traumatic, or infectious abnormalities of the lymphatic system cause severe complications including lymphedema, chylous ascites, chylothorax, and lymphatic vascular anomalies. Recent findings suggest that many of these abnormalities are related not only to changes in lymphatic fluid transport function, but also lymphatic regulation of immune responses.

## Lymphatic Function Is Variable and Can Regulate Immune Responses

Lymphatic function is highly variable clinically and modulated by numerous factors including chronic inflammation, tumors, external stimuli such as radiation, age, obesity, and metabolic dysfunction. For example, reports published in the late 1990s showed that aging results in structural changes in the lymphatic system including loss of elasticity, reduced smooth muscle coverage, decreased number of mesenteric collecting vessels, and decreased mesenteric lymphatic flow (22, 23). More recent studies have shown that aging results in ultrastructural changes in collecting lymphatics resulting in tissue degeneration and loss of extracellular matrix components, decreased expression of contractile and regulatory proteins, and increased lymphatic vascular permeability (24, 25). These structural changes, together with changes in gradients of eNOS, iNOS, and histamine significantly decrease aging lymphatic vessel contraction, interstitial fluid transport function, transport of pathogens to regional lymph nodes, and clearance of macromolecules from the central nervous system (26, 27). Similar changes in lymphatic function have been reported in obesity. For example, obesity results in structural and physiologic changes in the lymphatic system including increased lymphatic leakiness, decreased collecting vessel contractility, and decreased lymph node size and changes in lymph node architecture (2). Obese patients have decreased clearance of interstitial fluid as compared to lean individuals (28), obesity increases the risk of developing lymphedema after surgery (29), and severe obesity can lead to the spontaneous development of lymphedema (30). Interestingly, obesity induced lymphatic abnormalities decrease adaptive immune responses and are reversible with treatments that promote lymphangiogenesis and increase lymphatic transport (31). These findings are important because they suggest that common comorbid conditions have significant effects on the lymphatic system and these changes in turn significantly modulate immune responses.

Variability in lymphatic function resulting from aging, obesity, or metabolic syndrome may play a key role in immune responses to solid tumors and provide a rationale for the fact that these comorbid conditions increase the risk of tumor development and metastasis. Solid tumors such as melanoma and breast cancer are surrounded by abnormal, leaky lymphatics with impaired lymphatic transport function. Tumor, draining lymph node lymphangiogenesis and increased VEGF-C expression by inflammatory cells increase tumor growth and metastasis. Lymphatic vessel density and VEGF-C expression correlates with cytotoxic T cell infiltration and expression of immunosuppressive factors (iNOS, IDO, Arg-1) in patients with melanoma indicating a possibility of LECs playing a dual role in promoting and hindering anti-tumor responses (32). These changes are associated with increased risk of local/regional tumor recurrence and decreased survival. Intradermal implantation of melanoma in mice that lack dermal lymphatics due to transgenic expression of K-14 VEGFR3-Ig results in more rapid tumor growth locally, decreased distant metastasis, and decreased inflammatory cell infiltration, and impaired dendritic cell migration to regional lymph node (33). Interestingly, K-14-VEGFR3-Ig mice had impaired tumor specific immune responses after vaccination. Lymphatic endothelial cell (LEC) presentation of tumor antigens on major histocompatibility complex proteins (MHCI or MHCII) in the context of PD-L1 (checkpoint molecule programmed death ligand 1) and the absence of co-stimulatory molecules results in suppression of T cell mediated immune responses by decreasing T cell activation and proliferation and increasing apoptosis (4, 34). Taken together, these findings suggest that tumor lymphatics regulate tumor immune response and modulate the tumor microenvironment (35).

## Lymphedema Results in Chronic Inflammation

Lymph node dissection for cancer treatment is the most common cause of lymphedema development in Western Countries. Because lymphedema in this scenario develops secondary to surgical injury, this type of lymphedema is referred to as secondary lymphedema. Patients with secondary lymphedema develop progressive fibroadipose deposition in the affected limb and have an increased risk of developing infections and secondary malignancies. These pathologic changes cause significant morbidity and decrease quality of life (36). It is estimated that 20–40% of patients who undergo treatment for solid malignancies such as breast cancer, melanoma, gynecological or urologic tumors, or sarcomas go on to develop lymphedema (37). Because these cancers are common, there is a large number of patients who are diagnosed with lymphedema annually. This fact, together with the fact that lymphedema is a life-long disease and survival following cancer treatment has significantly improved, is responsible for the increasing number of patients who suffer from lymphedema. Although estimates of the number of patients who suffer from secondary lymphedema are variable and range between 5 and 10 million individuals, it is important to note that even the most conservative estimates make lymphedema among the most common chronic disorders and the most common long-term complication of cancer treatment.

Historically, development of lymphedema has been thought to be secondary to impaired development of collateral lymphatics that bypass the zone of injury. Indeed, this concept led to the multiple preclinical studies reporting on the use of exogenous lymphangiogenic growth factors as a therapeutic treatment for lymphedema (38–40). However, more recent studies have shown that although abnormal collateral lymphatic formation is a pathologic finding in patients with lymphedema, the clinical development of lymphedema may not be due to impaired production of lymphangiogenic cytokines such as VEGF-C (41, 42). In fact, patients with lymphedema have increased serum levels of VEGF-C (43) and transgenic mice that over-express VEGF-C have more severe pathologic changes of lymphedema in a tail model (44). These findings suggest that abnormalities in lymphangiogenesis alone are not enough to cause lymphedema. Rather, lymphatic injury appears to serve as an initiating factor setting into motion other pathologic changes that in some patients results in the development of lymphedema. This hypothesis is supported by the fact that lymph node dissection does not always cause lymphedema; instead only a subset of patients (about 1 in 3) who undergo this treatment go on to develop the disease. Further, the hypothesis that lymphatic injury is simply an initiating event that is necessary but alone insufficient to cause lymphedema is supported by the fact that the development of lymphedema in most cases occurs in a delayed fashion. Typically, patients who undergo lymph node dissection have minor swelling that resolves spontaneously 2–6 weeks after the initial surgery. In some patients however, this swelling recurs permanently 8–24 months later.

Recent studies from our lab and others have shown that lymphatic injury results in chronic inflammatory changes in the skin distal to the zone of injury and that this response, in turn regulates development of lymphedema by causing lymphatic leakiness, decreasing lymphatic pumping, increasing tissue fibrosis, and impairing development of collateral lymphatics. These inflammatory changes illustrate the important coordination of immune responses by the lymphatic system. The changes in inflammatory responses after significant lymphatic injury during surgery enable us to study the effects of more subtle forms of lymphatic injury as may occur in aging, obesity, metabolic dysfunction, or the tumor microenvironment. Thus, studying lymphedema is broadly relevant and may provide important insight into the role of the lymphatic system in regulating immune responses in other physiologic or pathologic events.

## Lymphatic Injury Results in Upregulation of Endogenous Danger Signal Molecules

Danger-associated molecular patterns (DAMPs) are the endogenous cellular products released by stressed, damaged or cells undergoing necrosis that alarm and activate the innate immune system components. By activating innate immune system, DAMPs create a pro-inflammatory state in the damaged tissues with an intention of host defense. However, in excess DAMPs can be harmful due to continuous activation of innate immune reactions (45, 46). Earlier studies by our group revealed the spatial and temporal expression patterns of High mobility group box 1 (HMGB1) and Heat shock protein 70 (HSP-70), two of the well-studied DAMPs (47). Using a mouse tail model of lymphedema tissues and human lymphedema biopsy samples, these studies have shown that DAMP expression occurred along a spatial gradient relative to the site of injury with the highest expression occurring closest to the zone of lymphatic injury and decreasing more distally. DAMP expression was localized to virtually all tissue cells including LECs, blood endothelial cells, adipocytes, and other stromal cells. More importantly, the expression of DAMPs persisted chronically even 6 weeks post-surgery, a time period that is far longer than wound healing related to the initial surgery. Other studies have shown that HMGB1 promotes lymphangiogenesis *in vivo* and *in vitro* (48, 49). In support of this, we found that blockade of HMGB1 in the mouse tail lymphedema model inhibited inflammatory lymphangiogenesis.

DAMPs initiate innate immune responses by interacting with pattern recognition receptors (PRRs) such as Toll-like receptors (TLRs). To understand the role of DAMPs interaction with PRRs in lymphedema development, we previously studied lymphedema development in different TLR knockout mice (TLR 2, 4, and 9 KO) using a mouse tail model of the disease (47). Consistent with our findings with HMGB1 blockade resulting in impaired lymphangiogenesis, we found that TLR knockout mice had more severe lymphedema, decreased lymphatic transport, abnormal lymphatic structures, decreased number of lymphatic capillaries, increased collagen deposition and dermal fibrosis, and increased infiltration of T cells as compared with wild-type controls. Taken together, these studies indicate that lymphatic injury chronically activates DAMPs, that eventually activates TLRs. The net result of this DAMP-TLR cascade activation during lymphatic injury is regulation of inflammatory lymphangiogenesis and chronic inflammatory reactions.

## Role of Macrophages During Lymphatic Injury and Lymphedema Progression

In similar lines with several other inflammation pathologies, macrophage recruitment and accumulation is significantly observed during lymphedema both in human biopsy (50, 51) and animal lymphedema models (50, 52). It is reported that macrophage recruitment is significantly high immediately after lymphatic injury compared to later stages of lymphedema (53). Macrophages seem to play multiple roles in lymphedema pathology based on results from several groups. Studies from our group shows that depletion of macrophages promotes impaired lymphatic function, infiltration of CD4^+^ cells and aggravates fibrosis (50). In addition we have demonstrated that during lymphatic injury, M2-differentiated macrophages are responsible for initiation of superficial dermal lymphangiogenesis by secreting lymphangiogenic growth factors like VEGF-C (54). Furthermore, it is being reported that, macrophage induced VEGF-C production is positively influenced by prostaglandin E2 (55) or CD4^+^ T cells and blocking of COX2, IFN-γ or IL-17 abrogates VEGF-C expression by macrophages (52) indicating interplay between inflammatory mediators and T cells with macrophages during lymphedema. Macrophages play a double-edged sword role in lymphedema pathology, because it plays important role in initial lymphangiogenesis post-lymphatic surgery transiently alleviating fluid accumulation (54). However, macrophages strongly express iNOS and are potential source of nitric oxide (NO) which attenuates collecting lymphatic vessels contraction and pumping significantly decreasing lymphatic function and eventually accumulation of lymph fluid and immunosuppression (9). Macrophages are also a major source of IL-6, a cytokine implicated in mediating chronic inflammation and adipose metabolism in lymphedema and found abundantly in lymphedema tissues (56, 57). Furthermore, macrophages are important source of TGF-β a major anti-lymphangiogenic cytokine that inhibits lymphangiogenesis and is copiously present in lymphedema tissues (58, 59). Taken together, these studies suggest that macrophages play a complex paracrine role in pathology of lymphedema regulating lymphangiogenesis, fibrosis and lymphatic function mostly through varied kinds of growth factor and cytokine secretion that have a dual impact on lymphatic endothelial cells.

## Lymphatic Injury Results in Activation of Dendritic Cells in the Skin and Migration to Regional Lymph Nodes

**Figure 1:**
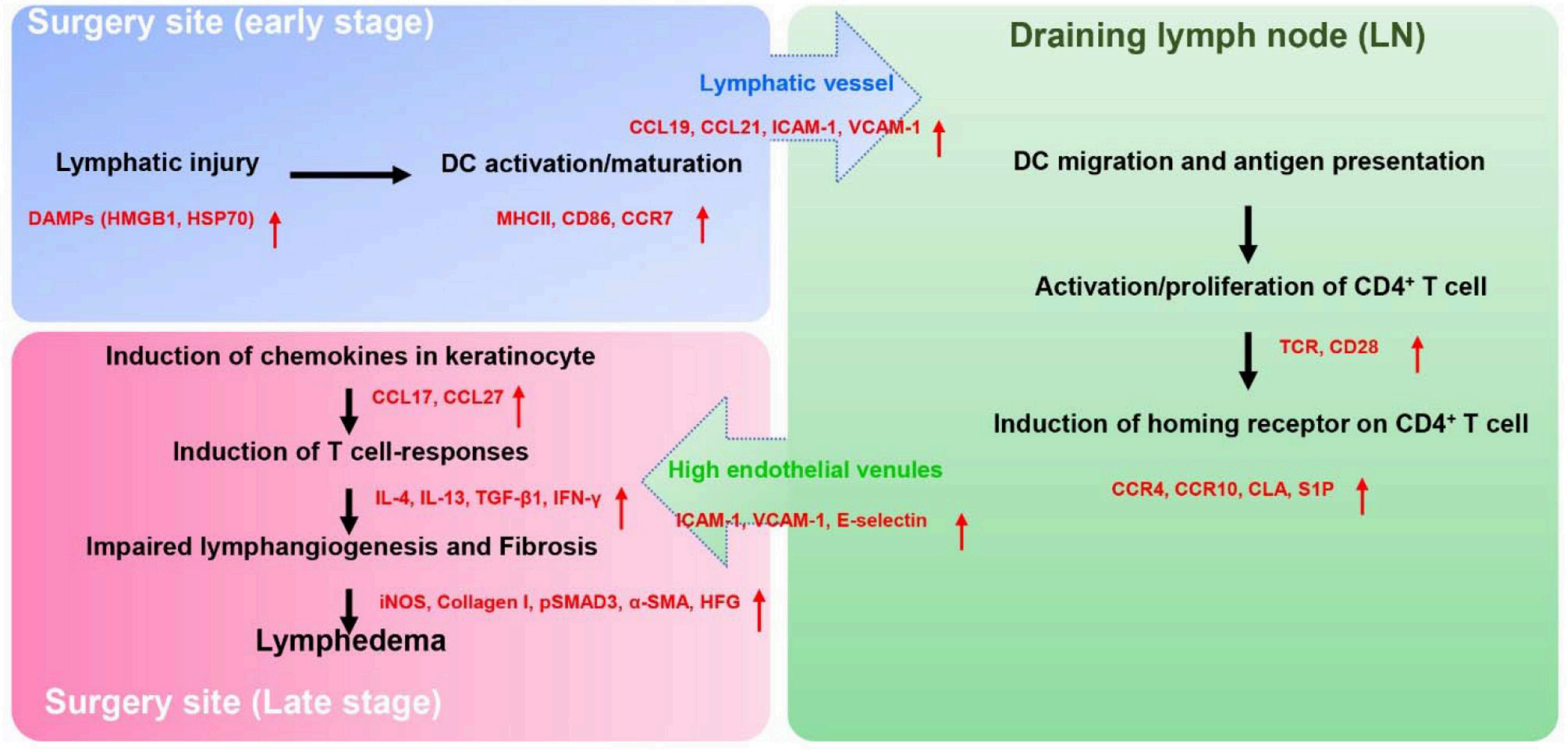
The proposed pathophysiology of secondary lymphedema. Lymphatic injury results in activation of DCs and maturation of DCs. Activated DCs migrate to skin draining lymph node(s) through the interaction of their chemokine receptors (CCR7) and chemokine (CCL19, CCL21) produced by LECs. Within the lymph node, activated DCs interact via T cell receptors and co-stimulatory molecules (CD28) with naïve CD4^+^ T cells resulting in T cell activation and Th1/Th2 differentiation. Activated CD4^+^ T cells express skin homing receptors (CCR4, CCR10, and CLA), are actively released from the lymph node into the systemic circulation, and home preferentially migrate to the skin in the area of lymphatic injury following gradients of CCL17, CCL27 which are mainly produced by keratinocyte. Upon the arrival in the skin, activated CD4^+^ T cells produce the Th1 (IFN-γ) or Th2 inflammatory mediators (IL-4, IL-13, TGF-β1), which promote lymphedema development by causing fibroadipose tissue deposition, impair lymphangiogenesis, decreased lymphatic pumping, increased lymphatic leakiness, and chronic inflammation.

How do chronic inflammatory responses in lymphedema get initiated? We have studied this question using mouse models of lymphedema and adoptive transfer of labeled cells to track the homing, activation, and migration of inflammatory cells (60). Adoptive transfer using intravenous injection is useful tool since this approach can provide insight into the behavior of circulating and skin resident inflammatory cells. To study activation of chronic T cell responses, we injected labeled dendritic cells (DCs) since these leukocytes are powerful antigen presenting cells that regulate adaptive T cell inflammatory responses. Wild-type or CD4 knockout mice (CD4KO) underwent popliteal lymph node dissection (PLND) and allowed to recover. Two weeks following surgery, we adoptively transferred bone marrow DCs using intravenous injection and analyzed DCs in the skin and the inguinal lymph node (the next draining basin) using flow cytometry. Interestingly, we found that adoptively transferred DCs rapidly migrated to the lymphedematous skin (within 6 h of injection) where they expressed activation markers. Over the next 24 h, activated DCs migrated to the inguinal lymph node. Importantly, DC activation or migration was identical in wild-type and CD4KO mice suggesting that DC activation precedes chronic CD4^+^ cell inflammatory reactions in lymphedema. These findings are supported by previous studies demonstrating that the lymphatics play a key role in regulating DC migration. Activated DCs upregulate cell surface expression of the chemokine receptor CCR7 (C-C chemokine receptor 7) whose ligands [CCL21 (C-C motif ligand 21) and CCL19] are expressed by LECs. Gradients of CCL21 guide DCs to initial lymphatics (61) and docking to CCL21 (62), and adhesion molecules such as intracellular adhesion molecule 1 (ICAM1), vascular cell adhesion molecule (VCAM1) expressed by LEC is required for entry into the vessel lumen (63). DCs enter the lymphatic vessel through gaps between LECs (64) and are guided to lymph nodes by gradients of CCL21 in lymphatic fluid (65) as well as passive lymphatic fluid flow (Figure 1).

## CD4^+^ Cell Inflammatory Responses Are Necessary for the Development of Lymphedema

DCs activate naïve T cell in lymph nodes by presenting antigens in the context of co-stimulatory molecules. This process is also important for chronic T cell inflammatory reactions in lymphedema. Histological and flow cytometry analysis of tissue biopsies from patients with lymphedema as well as mouse models of lymphedema demonstrate that the predominant inflammatory cell infiltrate is comprised of CD4^+^ cells. In fact, the severity of lymphedema correlates significantly with the degree of CD4^+^ cell inflammatory response. T cell inflammatory responses are necessary for the development of lymphedema since nude mice (lack all T cells) or mice lacking CD4^+^ cells (CD4KO) do not develop lymphedema after skin and lymphatic excision. Similarly, depletion of T cells using antibodies or topical treatment with tacrolimus, a medication that prevents T cell proliferation/differentiation prevents development of lymphedema in preclinical models and can be used to treat the disease once it has developed. In contrast, depletion of other inflammatory cell types such as cytotoxic T cells, B cells, and macrophages either has no significant effect or worsens the severity of lymphedema (66).

## T Cell Activation in Lymphedema Requires T Cell Receptor Activation and Co-Stimulatory Molecule Expression

T cell activation and differentiation in lymphedema requires T cell receptor activation in the context of co-stimulatory molecule expression by antigen presenting cells in regional lymph nodes (67). To demonstrate this concept, we used the adoptive transfer experimental approach to study T cell activation in lymphedema. In these experiments, CD4KO mice underwent PLND and 2 weeks later were intravenously injected with naïve CD4^+^ cells harvested from wild-type mice or RAG2/OTII mice since these transgenic mice have clonal T cells that only express the ovalbumin T cell receptor (68). This experiment was therefore designed to determine if T cell receptor activation is necessary for skin homing of activated T cells in lymphedema. In other experiments, we tested the hypothesis that T cell receptor activation requires co-stimulatory molecule expression by antigen presenting cells by adoptively transferring naïve wild-type CD4^+^ cells into either wild-type or transgenic mice lacking CD28 (a costimulatory molecule necessary for full T cell activation) (69). In these experiments, in contrast to our findings with adoptive transfer of DCs, we found that naïve T cells initially migrated to the ipsilateral inguinal lymph node (i.e., the next draining lymph node basin downstream from where the initial lymph node dissection was performed). Within the lymph node, both T cell receptor activation and co-stimulatory molecule activation were necessary for activation of CD4^+^ cells following lymphatic injury. Thus, adoptively transferred CD4^+^ cells harvested from RAG2/OTII mice were not activated in the inguinal lymph node and did not migrate to the lymphedematous skin. Similarly, adoptive transfer of wild-type CD4^+^ cells to CD28 knockout mice failed to result in T cell activation or T cell homing. Taken together, these findings suggest that T cells in lymphedema are activated in regional lymph nodes by antigen presenting cells in response to antigenic stimuli (60). Identification of T cell activating antigens in lymphedema is an active topic of study in our lab (Figure 1).

## Lymphedema Results in a Mixed T Helper Cell Differentiation Response

Lymphatic injury results in a mixed T cell inflammatory reaction consisting of T helper 1 (Th1), T helper 2 (Th2), and Tregs (66). Lymphedematous skin from clinical biopsy specimens and mouse models of lymphedema are infiltrated with large numbers of CD4^+^ cells that co-express interferon gamma (IFN-γ; putative Th1 cells) and CD4^+^ cells that co-express interleukin 4 (IL4) or IL13 (putative Th2 cells). T cells in lymphedematous tissues tend to cluster around initial lymphatics and lymphatic collectors (60, 70). Using adoptive transfer experiments, we found that naïve CD4^+^ cells are activated in regional lymph nodes and characterized by cell surface expression of Th1 (CD45^+^/CD4^+^/CCR5^+^/CXCR3^+^) and Th2 (CD45^+^/CD4^+^/CCR4^+^/CCR8^+^) cells. More importantly, we found that release of activated T cells from the lymph node via sphingosine 1 phosphate (S1P) signaling into the systemic circulation is necessary for the development of lymphedema. Treatment with an S1P inhibitor (FTY720) prevented release of activated T cells from the lymph node and prevented development of lymphedema in a mouse tail model of the disease.

Once activated T cells are released from the lymph node and actively home to lymphedematous skin by expressing skin homing cell surface receptors (71). CD4^+^ cell migration to the skin in other inflammatory conditions is regulated by cell surface expression of chemokine receptors including cutaneous leukocyte antigen (CLA), cc chemokine receptor 4 (CCR4), CCR8, and CCR10 (72, 73). This fact, together with the finding that T cell inflammatory reactions are important regulators of lymphedema, suggests that the expression of skin homing receptors may also play an important role in the development of lymphedema. This hypothesis is supported by the finding that the expression of CLA ligand E-selectin (74) as well as other leukocyte adhesion molecules (ICAM1, VCAM1) is significantly increased in lymphedematous skin (60). Similarly, we have found that the expression of ligands for CCR4 [chemokine c-c motif ligand 17 (CCL17)] and CCR10 (CCL27) (75) is markedly increased in keratinocytes of lymphedematous skin. Thus, migration of activated T cells to lymphedematous skin is not random but rather a tightly coordinated active process that may enable us to design rational treatment options that may be useful for the treatment of this morbid disease (Figure 1).

## Th2 Differentiation Is Necessary for Pathologic Changes in Lymphedema

Lymphedema is characterized by fibro-adipose tissue deposition, impaired lymphatic pumping, lymphatic leak, and decreased formation of collateral lymphatics. Previous studies in our lab and others have shown that T cells in general, and Th2 cells in particular play a key role in these pathologic processes (52, 60, 66, 70, 76). In fact, we have hypothesized that lymphedema is simply fibrotic organ failure of the lymphatic system. This hypothesis is supported by the histological characteristics of lymphedema demonstrating progressive collagen deposition and encasement of initial lymphatics by thick collagen bundles (66, 77). In addition, clinical studies have shown that late stage lymphedema results in fibrosis of collecting lymphatics with resultant luminal obliteration and failure of the pump mechanism (78, 79). The fibrotic hypothesis of lymphedema also provides a rationale for the delayed onset of symptoms following surgery since the critical threshold of fibrosis necessary to become symptomatic takes time to occur. In addition, fibrosis is a common cause of organ failure affecting virtually every other organ system in one form or another. Similar to lymphedema, these diseases are progressive and eventually become irreversible with severe end organ injury.

Previous studies have shown that T helper cells play a key role in organ fibrosis in a variety of pathologic conditions including liver fibrosis, pulmonary fibrosis, and scleroderma (80–82). Although the inciting events causing fibrosis in these conditions is highly variable and the parenchyma in these organ systems is distinct, the cellular mechanisms that regulate fibrosis in these conditions appears to be conserved and dependent on chronic Th2 biased immune responses. Ordinarily, Th2 cells play an important role in responses to parasites, however, chronic Th2 biased inflammatory responses promote tissue fibrosis by increasing collagen deposition, decreasing collagen breakdown, and increasing expression of profibrotic growth factors such as IL4, IL13, and TGF-b1 (83–85). Because Th1 immune responses often balance and oppose Th2 responses, in general Th1 biased responses are anti-fibrotic.

The regulation of organ fibrosis by chronic mixed Th1/Th2 inflammatory responses is referred to as the Th1/Th2 paradigm (86) and also appears to play a key role in the development of lymphedema. This hypothesis is supported by the fact that inhibition of Th2 differentiation with antibodies that neutralize IL4 or IL13, cytokines necessary for naïve T helper cell differentiation along the Th2 lineage, is highly effective in preventing the development of lymphedema in mouse models (70). Similarly, this treatment strategy is effective in reversing lymphedema once it has become established. Mice with impaired Th2 differentiation capacity do not develop lymphedema following lymphatic injury; in contrast, mice with impaired Th1 differentiation have a phenotype that is indistinguishable from wild-type littermates (71). Inhibition of Th2 differentiation markedly decreases accumulation of inflammatory cells in the skin, decreases collagen deposition and lymphatic fibrosis, reduces lymphatic leakiness, and preserves collecting lymphatic pumping capacity. Inhibition of Th2 responses decreases accumulation of perilymphatic inflammatory cells and markedly decreases expression of induced nitric oxide (iNOS) by perilymphatic inflammatory cells. This is important since increased iNOS expression in inflammatory conditions decreases lymphatic pumping capacity by decreasing gradients of endothelial derived nitric oxide expression by lymphatic cells (9). Finally, we have shown that T cell derived cytokines including IFN-γ, IL4, IL13, and TGF-β1 have potent anti-lymphangiogenic activity and impair LEC proliferation, differentiation, and migration (59, 87–89). Thus, Th2 mediated inflammatory responses impair lymphatic function by multiple mechanisms and play a central role in the pathology of lymphedema. More importantly, we have shown that other causes of lymphatic injury such as high fat diet induced obesity have a similar phenotype including peri-lymphatic accumulation of inflammatory cells, decreased lymphangiogenesis, lymphatic leaking, and impaired lymphatic pumping suggesting that our findings in lymphedema have broader physiologic relevance (90, 91).

Taken together, our findings in lymphedema suggest that lymphatic injury results in a mixed Th1/Th2 immune response secondary to T cell receptor mediated interactions with dendritic cells in regional lymph nodes and that these activated T cells migrate specifically to lymphedematous skin due to expression of cell surface receptors. Within the skin, Th2 cells proliferate and regulate pathologic changes including fibrosis, lymphatic leakiness, impaired pumping, and decreased formation of collateral lymphatics that eventually result in lymphedema.

## How Does Lymphatic Injury Regulate T Regulatory Cell Proliferation and Differentiation?

Tregs are immune cells that play a central role in regulating inflammatory responses, autoimmunity, and immune tolerance in a wide variety of physiologic settings. Tregs inhibit immune responses by a myriad of mechanisms including regulation of immune cell proliferation, apoptosis, and activation, production of cytokines, prevention of co-stimulation, and uptake of interleukin 2 (92, 93). These responses provide a homeostatic mechanism that prevents excessive inflammatory reactions. Tregs can be broadly divided into induced Tregs or natural Tregs; natural Tregs develop in the thymus from bone marrow derived T cell precursors (94). Induced Tregs, in contrast, develop from mature conventions T helper cells outside of the thymus, play an important role in the regulation of autoimmunity.

In addition to a mixed Th1/Th2 immune response, our lab and others have shown that lymphedema results in the accumulation of Tregs in lymphedematous tissues (66, 95, 96). Biopsy specimens of patients with unilateral upper extremity lymphedema demonstrated a nearly 6-fold increase in the number of Tregs in the lymphedematous skin (95). Using a mouse model of axillary lymph node dissection (ALND), we showed that the majority of Tregs present in the forelimb skin distal to the zone of lymphatic injury are proliferating, natural Tregs (CD4^+^/FoxP3^+^/Nrp-1^+^). In contrast, we found no changes in the number of induced Tregs in the skin and no changes in any Treg population in the blood or the spleen suggesting that Treg activation and proliferation was localized to the forelimb skin rather than systemic changes. Depletion of Tregs using diphtheria toxin treatment in Fox-P3-diphtheria toxin receptor (FoxP3-DTR) transgenic mice significantly increased the number of Th1 and Th2 cells in the forelimb tissues. In addition, this treatment increased the number of infiltrating macrophages (CD11b^+^F4/80^+^), neutrophils (Ly-6G^+^), and activated DCs (CD11c^+^MHCII^+^CD86^+^). Consistent with the immunosuppressive effects of Tregs in general, we found that Treg depletion improved T cell and B cell mediated immune responses after sensitization of the forelimb skin distal to the zone of lymphatic injury. Moreover, we found that Treg depletion increased bacterial phagocytosis and removal as compared with control mice after injection of heat inactivated bacterial particles in the forelimb skin. Other recent studies have shown that depletion of Tregs in mouse models of lymphedema results in increased severity of lymphedema while adoptive transfer of Tregs ameliorates the phenotype (96). Taken together, these findings suggest that Treg infiltration following lymphatic injury acts to suppress chronic inflammatory responses and may be homeostatic in nature. In addition, chronic infiltration of Tregs in lymphedematous tissues and subsequent suppression of immune responses may provide a rationale for the increased risk of infections and developing secondary malignancies in patients with lymphedema. These two papers in combination reveal the duality in Tregs function in lymphedema pathology by modulating one common factor namely inflammation. More importantly, these findings clearly show that lymphatic function can regulate Treg migration, proliferation, and differentiation.

## Conclusions

The lymphatic system, acting via direct and indirect mechanisms, is an important regulator of immune responses (Table 1). Lymphatic injury occurring either as a result of iatrogenic causes or secondary to physiologic changes such as obesity, tumor formation, metabolic syndrome, or infection can modulate immune response by regulating trafficking of antigen presenting cells, decreasing transport of particulate matter or antigens, regulating T cell differentiation, and modulating immunosuppressive immune responses. These changes may modulate the severity of the underlying condition and, in some cases, may promote the development of a vicious cycle of events. Thus, understanding the mechanisms regulating immune modulation by the lymphatic system is an important goal and has broad biologic relevance.

**Table 1:** The cellular and molecular factors in lymphedema development.

**Role in lymphedema development**	**Cell type**	**Mediator**	**Physiological function in lymphedema**	**References**
Promotion of lymphedema development	Dendritic cell	CCR7	- Migration of DC into lymph node	(61, 97, 98)
		MHCII, CD86	- T cell activation via antigen presentation	(60)
	Helper T cell	TCR, CD28	- T cell activation	(67–69)
		CCR4, CCR8, CCR10, CLA, S1P	- T cell homing to lymphedematous tissue	(72, 73, 75, 98)
	Th1 cell	IFN-γ	- Inhibition of lymphangiogenesis	(89)
	Th2 cell	IL-4, IL-13	- Th2 cells differentiation - Inhibition of lymphangiogenesis - Promotion of fibrosis	(59, 70, 88)
		TGF-β1	- Promotion of fibrosis	(59, 87, 88)
	Th17 cell	IL-17A	- Inhibition of lymphatic vessel formation	(52, 99)
	Lymphatic endothelial cell	CCL21, CCL19, ICAM-1, VCAM-1	- Migration of DC into lymph node	(61–63)
		eNOS	- Promotion of lymphatic vessel contraction	(9)
	Blood endothelial cell	ICAM-1, V-CAM1, E-selectin	- T cell homing to lymphedematous tissue	(60, 74)
	Macrophage	iNOS	- Inhibition of lymphatic collector contraction	(9, 90)
	Keratinocyte	CCL17, CCL27	- T cell homing to lymphedematous tissue	(75)
Inhibition of lymphedema development	Macrophage	VEGF-C, VEGF-A	- Promotion of lymphangiogenesis	(47, 50)
		IL-6	- Regulation of chronic inflammation and adipose metabolism	(56, 57, 100)
	Regulatory T cell	N.D.	- Inhibition of infiltration and activation of immune cell (Th1/Th2 cell, macrophage, neutrophils, activated DC)	(66, 95, 96)
Not involved in lymphedema development	Natural killer cell	N.D.	- Depletion of NK cells does not reverse lymphedema	(66)
	Cytotoxic T cell	N.D.	- Depletion of CD8^+^ cells depletion does not reverse lymphedema	(66)
	B cell	N.D.	- No significant differences in the percentage of B cells in mice model of lymphedema	(66)

*N.D. stands for not determined*.

## Author Contributions

BM, RK, JB, HP, and IW did the literature search, complied, and wrote the manuscript. SR and JS proof read the manuscript. RK and JB prepared the figures and tables.

### Conflict of Interest Statement

The authors declare that the research was conducted in the absence of any commercial or financial relationships that could be construed as a potential conflict of interest.
